# Peer Review of “Early Experience With Neutralizing Monoclonal Antibody Therapy for COVID-19: Retrospective Cohort Survival Analysis and Descriptive Study”

**DOI:** 10.2196/33499

**Published:** 2021-09-27

**Keywords:** infectious disease, monoclonal antibody therapy, COVID-19, experience, therapy, drug, patient outcome, risk, efficacy, approach, treatment, pandemic, antibody, immunotherapy, immune therapy

*This is a peer-review report submitted for the paper “Early Experience With Neutralizing Monoclonal Antibody Therapy for COVID-19: Retrospective Cohort Survival Analysis and Descriptive Study.”*

## Round 1 Review

### General Comments

In this paper [1], the authors study the effect of monoclonal antibodies and their benefit in patients with COVID-19. The authors conclude that, although this therapy may be an important treatment option for early mild to moderate COVID-19 in patients at high risk, further investigations are needed to define the optimal timing of monoclonal antibody treatment to reduce hospitalization and mortality.

Although this topic is not entirely new, the paper looks good to me and confirms other previously published data.

### Specific Comments

As Tables 1 and 2 are quite complex, they need a clear legend and not just the title, as reported.

For greater clarity, the figures should also be better explained.

In the Introduction and Discussion when talking about COVID-19, for the sake of clarity, we need to better explain the inflammation that kills people and not just go straight to the monoclonal antibodies. Therefore, to make this paper more interesting for the readers of this important journal, the authors should expand the discussion on this subject a little to give a wider view to the reader.
